# Occurrence, risk factors and genotypes of *Enterocytozoon bieneusi* in dogs and cats in Guangzhou, southern China: high genotype diversity and zoonotic concern

**DOI:** 10.1186/s12917-020-02421-4

**Published:** 2020-06-18

**Authors:** Haiyan Wang, Xuhui Lin, Yongxiang Sun, Nanshan Qi, Minna Lv, Wenwan Xiao, Yuancai Chen, Ruiping Xiang, Mingfei Sun, Longxian Zhang

**Affiliations:** 1 Key Laboratory of Innovation and Utilization of Unconventional Feed Resources, Henan University of Animal Husbandry and Economy, Zhengzhou, 450046 Henan People’s Republic of China; 2 Key Laboratory of Livestock Disease Prevention of Guangdong Province, Maoming Branch, Guangdong Laboratory for Lingnan Modern Agriculture, Scientific Observation and Experiment Station of Veterinary Drugs and Diagnostic Techniques of Guangdong Province, Ministry ofAgriculture, Institute of Animal Health, Guangdong Academy of Agricultural Sciences, Guangzhou, Guangdong Province 510640 People’s Republic of China; 3 College of Animal Science and Veterinary Medicine, Henan Agricultural University, Zhengzhou, 450046 Henan P. R. China

**Keywords:** Dogs, Cats, *E. Bieneusi*, Genotype, Inter-species, Zoonotic

## Abstract

**Background:**

*Enterocytozoon bieneusi*, a common opportunistic fungal pathogen, has a wide range of hosts. Limited epidemiological data on *E. bieneusi* intestinal infections in companion animals (dogs and cats) in China exists. In this study, fecal samples (651 from dogs and 389 from cats) in Guangzhou city, Guangdong Province, China, were collected, and the ribosomal internal transcribed (ITS) spacer region from the DNA extracted from them was Polymerase Chain Reaction (PCR)-amplified and sequenced.

**Results:**

Based on the sequencing data, *E. bieneusi* was identified in the fecal samples collected from 149 (22.9%) and 79 (20.3%) dogs and cats. Of the factors investigated, poor living conditions appeared to be the major risk factor for contracting the pathogen. Eleven *E. bieneusi* genotypes, six known (PtEb IX, GD1, D, CD9, EbpC, I) and five novel (designated here as GD2– GD6), were found in dogs. Eight genotypes, six known (PtEb IX, GD1, D, CD9, EbpC, Type IV) and two novel (GD2 and GC1), were identified in cats. Genotype PtEb IX was most common in both dogs and cats, followed by genotype GD1.

**Conclusions:**

Although PtEb IX was the most common *E. bieneusi* genotype in dogs, this is the first report of this genotype dominating in cats. The same genotype distribution of the pathogen between the two different companion animals species in the same geographic area indicates that inter-species transmission is probable. The widespread existence of zoonotic *E. bieneusi* genotypes (D, EbpC, Type IV) in companion animals indicates that they are potential sources of environmental contamination and infections in humans.

## Background

*Enterocytozoon bieneusi*, classified as fungi, is a well-known enteric microsporidia of animals and humans. *E. bieneusi* causes gastrointestinal diseases, such as self-limiting diarrhea, malabsorption, and can even be life-threatening, particularly in people with compromised immune systems (e.g., those with acquired immune deficiency syndrome (AIDS), organ transplantation recipients, children, and the elderly) [1]. The main transmission routes for contracting *E. bieneusi* infection are water and food-borne through its infective spores [2].

*E. bieneusi* is regarded as a zoonotic pathogen; however, the sources and transmission routes involved in infections in humans are unpredictable [2]. Genotyping and phylogenetic analysis of the internal transcribed spacer (ITS) of the ribosomal RNA gene (rDNA) can help with assessing *E. bieneusi* host specificity and zoonotic potential. To date, several hundred *E. bieneusi* ITS genotypes have been defined [3]. These genotypes cluster into the following different phylogenetic groups with different host specificities and public health significance: ITS genotypes in group 1 (e.g., D, type IV, EbpC), and sporadic genotypes (e.g., I, J, BEB4, Nig3–Nig5) in group 2, have been identified in humans and various other animals, and are of zoonotic concern. However, most of those genotypes in other groups (groups 3–11) are host-specific, and therefore of minimal public health significance [4, 5].

In the few molecular epidemiological surveys conducted on *E. bieneusi* in various animals to date, numerous genotypes have been detected. For example: D, EbpA, Type IV and EbpC in humans; D, CM1, Type IV, Peru11, EbpC and O in non-human primates; I, J, BEB4 and BEB6 in cattle; D, EbpA and EbpC in pigs; D, Type IV, Peru6 and I in rabbits; BEB6 and Peru6 in geese; EbpC, EpbA, horse1–3, and O in horses; PtEb IX, EbpC, D and Type IV in dogs; I, K and BEB6 in cats; D in wildlife [6–11]. However, as intimate companions of humans, dogs and cats have close contact with humans, but only the following Chinese regions have taken part in molecular epidemiological studies and assessments of zoonotic potential in these animals: Heilongjiang [12], Jilin [13], Henan [14], Anhui [15] provinces and Shanghai [16]. Guangzhou, southern China, is the third most economically-developed city in China, and boasts a large population of human residents (e.g., 111.69 million in 2017) and companion animals (e.g., there were > 10.62 million pets in the country in 2015) [17]. Here, we aimed to estimate the prevalence and genetic characteristics of *E. bieneusi* in dogs and cats living in the Guangzhou area, and assess the zoonotic potential between these companion animals and humans.

## Results

### *E. bieneusi* infection rates

PCR amplification of the ITS region detected *E. bieneusi* in 149 (22.9%; 95% CI: 22.9 ± 0.7%) of the 651 canine specimens, and 79 (20.3%; 95% CI: 20.3 ± 1.0%) of the 389 feline specimens. Risk factor analyses showed that the collection site had a significant effect on the risk of contracting an infection in both dogs and cats (Table 1). Animals from shelters (39.6% positivity for dogs, 27.7% positivity for cats) had higher infection rates than those from pet markets (36.4% positivity for dogs, 25% positivity for cats), pet hospitals (19.6% positivity for dogs, 12.9% positivity for cats), and breeding centers (11.4% positivity for dogs, 14.3% positivity for cats) (*P* < 0.01). Deworming had a strong protective effect on the risk of contracting an *E. bieneusi* infection, with non-dewormed dogs and cats having 2.6-times and 2.2-times higher risks of *E. bieneusi*-positivity, respectively, than dewormed animals (OR = 3.53 and 2.64, respectively, *P* < 0.01). The infection rates in juvenile animals (≤ 6 months of age) (26.1% positivity for dogs, 25.9% positivity for cats) were higher than those in adults (> 6 month of age) (19.3% positivity for dogs, 18.1% positivity for cats), but the differences were not significant (OR = 0.68 and 0.63, respectively, and *P* = 0.039 and 0.082, respectively). No significant difference was found between infection rates and sex in both dogs and cats (*P* = 0.313 and 0.897, respectively).

**Table 1:** Factors associated with the infection rate of *E. bieneusi* in dogs and cats in Guangzhou, China

Host	Factor	Category	No. tested	No. positive	% (95% CI)	OR (95% CI)	*P*-value
Dogs	Site	Shelters	149	59	39.6 (31.7–47.5)	Reference	*P <* 0.01
		Hospitals	199	39	19.6 (14.0–25.2)	0.37 (0.23–0.60)	
		Pet market	66	24	36.4 (24.4–48.3)	0.87 (0.48–1.59)	
		Breeding center	237	27	11.4 (7.3–15.5)	0.20 (0.12–0.33)	
	Age	≤ 6 month	345	90	26.1 (21.4–30.7)	Reference	0.039
		> 6 month	306	59	19.3 (14.8–23.7)	0.68 (0.47–0.98)	
	Sex	Male	361	88	24.4 (19.9–28.8)	Reference	0.313
		Female	290	61	21.0 (16.3–25.8)	0.83 (0.57–1.20)	
	Deworming	Yes	436	66	15.1 (11.8–18.5)	Reference	*P <* 0.01
		No	215	83	38.6 (32.0–45.2)	3.53 (2.41–5.15)	
Total			651	149	22.9 (19.7–26.1)		
Cats	Site	Shelters	141	39	27.7 (20.2–35.1)	Reference	0.010
		Hospitals	132	17	12.9 (7.1–18.7)	0.39 (0.21–0.73)	
		Pet market	60	15	25.0 (13.7–36.3)	0.87 (0.44–1.74)	
		Breeding centers	56	8	14.3 (4.8–23.7)	0.44 (0.19–1.00)	
	Age	≤ 6 month	112	29	25.9 (17.7–34.1)	Reference	0.082
		> 6 month	277	50	18.1 (13.5–22.6)	0.63 (0.37–1.06)	
	Sex	Male	229	46	20.1 (14.9–25.3)	Reference	0.897
		Female	160	33	20.6 (14.3–27.0)	1.03 (0.63–1.71)	
	Deworming	Yes	188	25	13.2 (8.5–18.0)	Reference	*P <* 0.01
		No	201	54	26.9 (20.7–33.0)	2.64 (1.54–4.51)	
Total			389	79	20.3 (16.3–24.3)		

*CI* confidence limit, *OR* odds ratio

### Distribution of *E. bieneusi* genotypes

Sequence analysis of the ITS region showed the presence of eleven *E. bieneusi* ITS genotypes, six of which are known (PtEb IX, GD1, D, CD9, EbpC and I) and five of which are novel (designated here as GD2–GD6) in 149 dogs, and eight genotypes, six of which are known (PtEb IX, GD1, D, CD9, EbpC and Type IV) and two of which are novel (GD2 and GC1) in 79 cats (Table 2). The most prevalent genotype in dogs, PtEb IX, which was identified in 56/149 positive specimens (37.6%), is identical to DQ885585 from a dog in Portugal (GenBank accession no. DQ885585). The second prevalent genotype, designated here as GD1, was identified in 38 specimens (25.5%). The GD1 nucleotide sequence is identical to a published porcine nucleotide sequence from China (GenBank accession no. MK705932), with one single nucleotide polymorphism (SNP) (T to C substitution at position 66) compared with the PtEb IX sequence (DQ885585). The novel GD2 genotype was identified in 24 isolates (16.1%), whereas the common D, EbpC and CD9 genotypes were seen in 12 (8.0%), 7 (4.7%) and 4 (2.7%) samples, respectively. Genotypes I, GD3 and GD4 were detected in 2 samples each (1.3%), whereas the remaining genotypes, GD5 and GD6, were identified in 1 sample each (0.7%). In cats, genotype PtEb IX (*n* = 25, 31.6%) was the most prevalent, followed by genotypes GD1 (*n* = 16, 20.2%) and D (*n* = 12, 15.2%). Genotype GD2 was found in 10 samples (12.7%), while Type IV, EbpC, GC1, and CD9, were observed in 6 (7.6%), 5 (6.3%), 3 (3.8%) and 2 (2.5%) samples, respectively. The nucleotide sequences from D, EbpC, I, CD9 and Type IV detected in this study, were identical to those deposited in GenBank under accession numbers MK478054, MK347522, MN178160, MN179310, and MK789441, respectively. Novel GD5 and GD6 genotypes each contained one SNP, comparable to genotype PtEb IX. Genotypes GD4 and GD2 each contained two SNPs comparable to genotypes PtEb IX and CD9, respectively. In contrast, genotypes GD3 and GC1 contained four SNPs comparable to genotype PtEb IX.

**Table 2:** Characteristics of *E. bieneusi* genotypes in dogs and cats in Guangzhou, China

Host	Factor	Category	No. positive	Genotype (n)
Dogs	Site	Shelters	59	PtEb IX (25), GD1* (28), D (6)
		Hospitals	39	PtEb IX (12), GD1* (10), GD2 (11), GD3 (2), GD4(2), GD5 (1), GD6(1)
		Pet market	24	PtEb IX (13), GD2 (5), D (6),
		Breeding center	27	PtEb IX (6), GD2 (8), EbpC (7), CD9 (4), I (2)
	Age	≤ 6 month	90	PtEb IX (45), GD1* (20), GD2^*^ (10), D (6), EbpC (4), GD3^*^ (2), GD4^*^ (2), GD5^*^ (1),
		> 6 month	59	PtEb IX (11), GD1* (18), GD2^*^ (14), D (6), EbpC (3), CD9 (4), I (2), GD6^*^ (1)
	Sex	Male	88	PtEb IX (41), GD1* (20), GD2^*^ (14), D (6), EbpC (3), GD3^*^ (2), GD4^*^ (1), GD5^*^ (1)
		Female	61	PtEb IX (15), GD1* (18), GD2^*^ (10), D (6), EbpC (4), CD9 (4), I (2), GD4^*^ (1), GD6^*^ (1)
	Deworming	Yes	66	PtEb IX (18), GD1* (10), GD2 (19), GD3 (2), GD4 (2), GD5 (1), GD6 (1), EbpC (7), CD9 (4), I (2)
		No	83	PtEb IX (38), GD1* (28), GD2 (5), D (12)
Total			149	PtEb IX (56), GD1* (38), GD2^*^ (24), D (12), EbpC (7), CD9 (4), I (2), GD3^*^ (2), GD4^*^ (2), GD5^*^ (1), GD6^*^ (1)
Cats	Site	Shelters	39	PtEb IX (7), GD1* (2), D (11), GD2* (10), Type IV (1), EbpC (5), GC1 (3),
		Hospitals	17	PtEb IX (6), GD1* (8), Type IV (2), D (1)
		Pet market	15	PtEb IX (8), GD1* (6), Type IV (1)
		Breeding centers	8	PtEb IX (4), Type IV (2), CD9 (2)
	Age	≤ 6 month	29	PtEb IX (8), D (1), GD1* (10), Type IV (2), EbpC (5), GC1 (3)
		> 6 month	50	PtEb IX (17), D (11), GD2* (10), GD1* (6), Type IV (4), CD9 (2)
	Sex	Male	46	PtEb IX (18), GD1* (7), GD2* (5), Type IV (4), CD9 (1), D (11)
		Female	33	PtEb IX (7), GD1* (9), GD2* (5), EbpC (5), GC1 (3), Type IV (2), CD9 (1), D (1)
	Deworming	Yes	25	PtEb IX (10), GD1* (8), D (1), Type IV (4), CD9 (2)
		No	54	PtEb IX (15), GD1* (8), D (11), GD2* (10), Type IV (2), EbpC (5), GC1 (3)
Total			79	PtEb IX (25), GD1* (16), D (12), GD2* (10), Type IV (6), EbpC (5), GC1(3), CD9 (2)

**Fig. 1:**
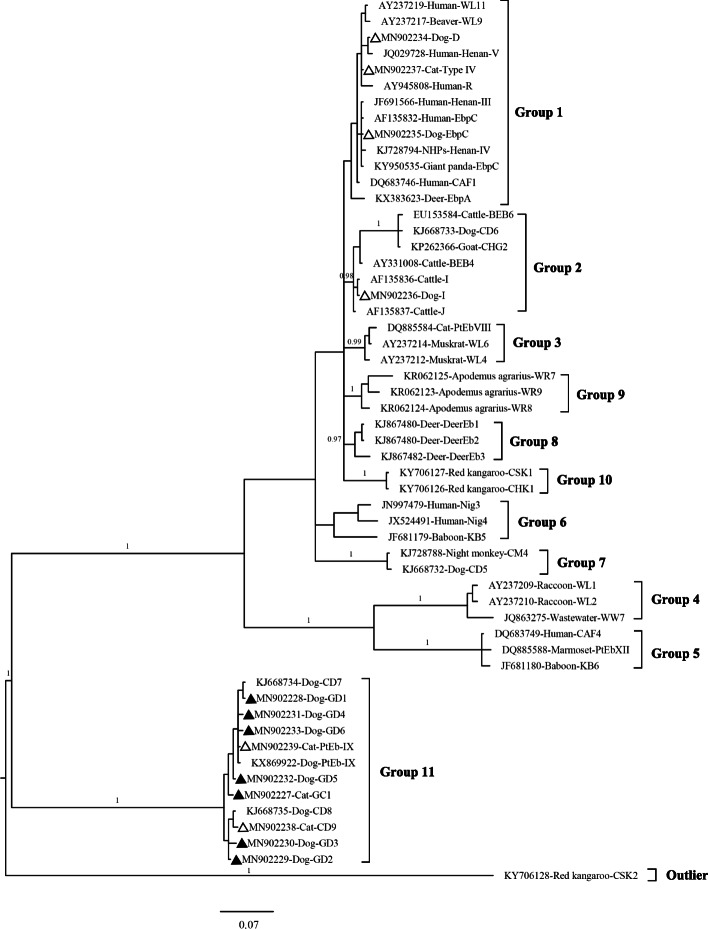
Phylogenetic tree for *E. bieneusi* based on Bayesian inference of ITS sequences. Statistically significant posterior probabilities are indicated on the branches. The *E. bieneusi* CSK2 (KY706128) genotype from red kangaroo was used as the outgroup. Known and novel genotypes identified in this study are indicated by triangles filled in white and black, respectively

Our phylogenetic tree based on Bayesian analysis of the sequences showed that the genotypes were classifiable into three clear types (D, EbpC, Type IV and I) within the previously designated zoonotic group 1and 2, whereas genotypes PtEb IX and CD9 clustered together with the other genotypes (GD1 to GD6 and GC1) into a dog-specific group 11 (Fig. 1).

## Discussion

Infections with *E. bieneusi* in dogs and cats have been reported in Spain, Brazil, China, Colombia, Germany, Japan, Portugal, Switzerland, Poland, Turkey, Iran, the Czech Republic, Egypt, and Thailand, where they range from 0 to 25.8% in dogs and from 1.4 to 31.3% in cats [7, 9, 14–16, 18–23]. In the present study, 149 dogs (22.9%) and 79 cats (20.3%) were *E. bieneusi*-positive by nested PCR-based sequencing of the ITS region, a somewhat higher rate than those reported in most previous studies. This discrepancy may be related to the fact that the fecal samples collected in our study included those from shelters and pet markets, where the dogs and cats raised together had poor living conditions. In contrast, the fecal samples from other studies were collected from households, veterinary clinics or pet shops where the animals were more likely to have lived in a clean environment [9, 15, 16, 19]. Thus, poor living conditions for dogs and cats may be a major risk factor for contracting *E. bieneusi* infections. Our risk analysis in the present study confirmed above conclusion as the infection rates in animals from shelters and pet markets were significantly higher than those from pet hospitals and breeding centers. There was no significant age-associated difference in *E. bieneusi* infection rates in dogs and cats for the age groups we studied (≤ 6 months juveniles vs. > 6 months adults). This finding accords with observations from Colombia, three surveys from Shanghai city and Henan and Anhui provinces in China [10, 14–16, 19], but differs from the findings from two recent Japanese and Australian studies where a noticeably significant correlation was shown between the occurrence of the pathogen and age of the dogs and cats [9, 24]. Similarly, two previous reports from Anhui and Henan provinces [14, 15] found no significant sex-associated difference in the occurrence of *E. bieneusi* and sex in dogs or cats. Not unexpectedly, deworming had a significantly negative effect on the risk of contracting *E. bieneusi* infections. But with no currently effective drugs against such infections [25], careful management of pet hygiene is very important. Nevertheless, various factors like host immunity, climate, and geography may contribute to *E. bieneusi* infection rates, as does the sample size used in epidemiological studies on this fungi.

A high degree of genetic diversity in *E. bieneusi* was observed in dogs (PtEb IX, GD1 to GD6, D, CD9, EbpC, I) and cats (PtEb IX, GD1 to GD2, D, CD9, EbpC, Type IV, GC1) in the present study; these findings show similarity with some studies in Heilongjiang, Henan and Anhui provinces, China [12, 14, 15]. In contrast, low genetic heterogeneity in *E. bieneusi* was observed in dogs and cats in other studies with only genotypes PtEb IX and D found in dogs and genotypes Type IV and D in cats in Shanghai city, China [16], only one genotype, PtEb IX, in dogs in Japan [24], and one genotype, D, in cats in Slovakia, Poland, and the Czech Republic [21]. As with most previous reports [10, 12, 14–16, 24], we identified genotype PtEbIX as dominant in dogs and cats. PtEb IX appears to have a relatively narrow host-range and is considered the most common dog-adapted *E. bieneusi* genotype [2]. However, this is the first time that genotype PtEb IX has been identified as dominant in cats, which is inconsistent with most studies that have reported that Type IV and D are most common in cats [7, 12, 14–16, 21, 26]. The same pathogen genotype distribution exists between companion animals in the same geographic area suggests that inter-species transmission of this pathogen likely occurs between dogs and cats in the study area.

Our sequencing data analysis revealed the presence of three known genotypes: D, EbpC and Type IV. This is in concordance with observations from some earlier studies in Brazil, Turkey, Japan, Thailand, Colombia, Germany, Portugal, Slovakia and China [7, 8, 12, 15, 16, 20, 21, 23, 26–28]. Genotypes D, EbpC, and Type IV are known to have a broad-host range and have been reported in nonhuman primates, dogs, cats and domestic animals, and even in wastewater [12, 15, 29–31]. Beyond this, these three genotypes are also known to have infected human immunodeficiency virus (HIV)-positive patients, AIDS patients and HIV-negative people in Henan [32]. Genotype D was also reported to have colonized renal transplant recipients in Spain [33] and a child in-patient at a Shanghai hospital in China [34]. Moreover, we detected genotype I in two dog samples. Genotype I has been documented in pigs, cattles, yaks, golden takins, deer, rabbits, macaques, cats, dogs, wild animals, and humans [6, 13, 35–38]. These studies support the zoonotic transmission and public health significance potential of the above-named genotypes. Thus, dogs and cats can serve as potential reservoir hosts for *E. bieneusi* transmission.

Our study also identified GD2 and CD9, two known *E. bieneusi* genotypes, and seven novel genotypes that displayed between 1 and 4 nucleotide differences, comparable with those of the dog-specific PtEb IX genotype [14]. Our phylogenetic analysis revealed that GD2, CD9 and the seven novel genotypes formed a separate cluster in the tree, featuring dog-adapted genotypes, thereby indicating their minimal public health importance.

## Conclusions

Our data indicate that dogs and cats in Guangzhou city are prone to harboring *E. bieneusi* infections, and their living conditions may play a major role in contracting this pathogen. We identified highly diverse *E. bieneusi* genotypes in the studied dogs and cats, which were mainly infected with the host-specific PtEb IX genotype, and this is the first report of this genotype being dominant in cats. The same genotype distribution in dogs and cats in the same geographic area suggests that inter-species transmission of this pathogen is probable within the study area. Nevertheless, the widespread prevalence of zoonotic genotypes (D, EbpC, Type IV and I) indicates that dogs and cats in Guangzhou city should be a public health concern. Further epidemiological data is needed if we are to fully understand the zoonotic transmission mechanism for microsporidiosis.

## Methods

### Sample collection

Altogether, 1040 stool samples (651 dogs, 389 cats) from shelters, hospitals, markets, and breeding centers for pets from eight different districts in Guangzhou city were collected between January 2018 and December 2018. Two shelters were located in the suburbs of Luogang and Huangpu districts where dogs and cats are raised together under poor living conditions. These animals originally roamed free in the nearby streets and were later found by local citizens and sent to the shelters. The markets selling pets are located in the urban area of Tianhe district, which is a crowded and moist environment. In contrast, the two breeding centers were in the suburbs of Conghua and Nansha districts where good living conditions and facilities prevail and from where high quality dog and cat breeds are supplied. All fresh fecal samples from the shelters, pet markets and breeding centers were collected once, directly from the floor of the cage or per-rectum. Care was taken to avoid sampling fecal material that had contacted the ground. Samples from the four pet hospitals in the urban area of Tianhe, Baiyun, Huadu, and Panyu districts were collected by each animal’s owners according to our instructions. Prior to sampling, these owners were randomly selected and provided consent for the use of samples from their animals in this study. All the fresh feces collected were from pets with no apparent clinical signs of infection. The samples were placed into clean plastic bags marked with ID numbers corresponding to the date, collection site, and the age, sex, and deworming status of the pets. The plastic bags were sealed and immediately placed on ice packs in an insulated container. Samples were transported to the laboratory, stored at 4 °C, and processed no later than 24 h after collection.

### DNA extraction

A 10-g aliquot of each sample was individually mixed with 30 ml of distilled water and passed through a wire mesh sieve of ~ 250 μm in width. Suspensions were centrifuged at 3000×g for 5 min and the precipitates were used for DNA extraction. Genomic DNA, which was extracted from 200 mg of each precipitate using the E.Z.N.A. Stool DNA Kit (Omega Bio-Tek Inc., Norcross, GA, USA) according to the manufacturer’s instructions, was stored at − 20 °C.

### PCR and genotyping

Each DNA specimen was used to test for the presence of *E. bieneusi* by targeting the ITS region of the rRNA gene (product size ~ 390 bp) as previously described [39]. Each 25 μl PCR mixture contained 0.4 μM of each primer, 2.5 μl 10× Ex Taq Buffer (Mg2+ free), 2 mM MgCl2, 0.2 mM dNTP mixture, 0.625 U of TaKaRa Ex Taq (TaKaRa Shuzo Co., Ltd), and 1 μl of genomic DNA. Each sample was analyzed in duplicate using positive (cattle-derived DNA) and negative (sterile water) controls.

Positive secondary PCR products were directly sequenced by GENEWIZ (Suzhou, China), and the sequence accuracy was confirmed by two-directional sequencing. The genotype identities of the *E. bieneusi* sequences were determined by comparing the sequences we obtained with reference sequences from the National Center for Biotechnology Information (https://www.ncbi.nlm.nih.gov/) database using Clustal X 2.1 (http://www.clustal.org) sequence alignments.

### Phylogenetic analysis

Phylogenetic trees were constructed by Bayesian inference with Monte Carlo Markov Chain methods in MrBayes v 3.2.6 (http://mrbayes.sourceforge.net/). Nucleotide substitutions were determined using the general time reversible model (GTR + G) in Model Test version 3.7 (http://www.molecularevolution.org/). Statistically significant posterior probabilities are indicated at branches, and greater than > 95% was shown on nodes.

### Statistical analyses

Differences in parasite prevalence based on the collection site, age, sex, and deworming status were compared using a χ2 test in SPSS version 22.0 for Windows by variable analyses (SPSS Inc., Chicago, IL, USA). Differences with *p*-values of < 0.01 were considered significant. Additionally, 95% confidence intervals (95% CIs) and odds ratios (ORs) were calculated to explore the strength of the association between *E. bieneusi* positivity and the factors tested.

## Data Availability

The datasets analyzed during the current study are available in the NCBI GenBank repository (https://www.ncbi.nlm.nih.gov/genbank/) under accession numbers MN902227-MN902239.

## Abbreviations

*E. bieneusi*: *Enterocytozoon bieneusi*
ITS: Internal transcribed spacer
PCR: Polymerase Chain Reaction
AIDS: Acquired immune deficiency syndrome
HIV: Human immunodeficiency virus
